# Awareness of wearing an accelerometer does not affect physical activity in youth

**DOI:** 10.1186/s12874-017-0378-5

**Published:** 2017-07-11

**Authors:** Jérémy Vanhelst, Laurent Béghin, Elodie Drumez, Stéphanie Coopman, Frédéric Gottrand

**Affiliations:** 1 Univ. Lille, Inserm, CHU Lille, U995 – LIRIC – Lille Inflammation Research International Center, CIC 1403 – Centre d’investigation clinique, F-59000 Lille, France; 2 Univ. Lille, CHU Lille, EA 2694 – Public Health: epidemiology and quality of care, F-59000 Lille, France

**Keywords:** Young, Activity monitor, Free living conditions, Reactivity

## Abstract

**Background:**

This study aimed to investigate whether awareness of being monitored by an accelerometer has an effect on physical activity in young people.

**Methods:**

Eighty healthy participants aged 10–18 years were randomized between blinded and nonblinded groups. The blinded participants were informed that we were testing the reliability of a new device for body posture assessment and these participants did not receive any information about physical activity. In contrast, the nonblinded participants were informed that the device was an accelerometer that assessed physical activity levels and patterns. The participants were instructed to wear the accelerometer for 4 consecutive days (2 school days and 2 school-free days).

**Results:**

Missing data led to the exclusion of 2 participants assigned to the blinded group. When data from the blinded group were compared with these from the nonblinded group, no differences were found in the duration of any of the following items: (*i*) wearing the accelerometer, (*ii*) total physical activity, (*iii*) sedentary activity, and (*iv*) moderate-to-vigorous activity.

**Conclusions:**

Our study shows that the awareness of wearing an accelerometer has no influence on physical activity patterns in young people. This study improves the understanding of physical activity assessment and underlines the objectivity of this method.

**Trial registration:**

NCT02844101 (retrospectively registered at July 13th 2016).

## Background

Physical activity (PA), especially moderate-to-vigorous PA (MVPA), is widely recognized as an important determinant of health in children and adolescents [1, 2]. Thus, accurate measurement of PA is essential for developing intervention strategies in epidemiological studies. PA questionnaires, diaries, pedometers, and accelerometers have been used widely to assess PA in free-living conditions [3].

Accelerometry is frequently used in PA studies and it is recognized as a reliable, valid, and objective measurement [4]. A major concern regarding the objective assessment of PA is the Hawthorne effect, i.e. the change in PA behavior related to the participant’s awareness of being monitored to actually assess their PA pattern [3, 5]. The term *reactivity* has been used to describe the action of modifying PA behaviors when wearing a measurement device [3, 6, 7]. In other words, participants in PA studies may increase their daily and routine activities when they know that they are wearing a device to assess their habitual PA.

Findings on the reactivity measured with a pedometer in children and adolescents are controversial; however, few studies have assessed reactivity using accelerometer devices in youth [6, 8–12]. One study showed no reactivity to accelerometers in young adults in contrast to another study in teenagers who were all well-informed about the nature and purpose of the device (i.e. it measured their PA patterns), which may have elicited some degree of reactivity [6, 13]. Recently, Davis and Loprinzi did not observe sufficient evidence of accelerometer reactivity among United States (US) children and adolescents [8].

The conflicting results of the previous studies on accelerometer reactivity in youth create difficulties in the interpretations and understanding of accelerometer reactivity in young people [6, 8]. Moreover, the authors used an indirect methodology (i.e. a nonexperimental design) that could lead to a potential bias in their results.

Therefore, the aim of this study was to assess reactivity to an accelerometer in young people by comparing PA measured in teenagers wearing the device who were randomized into 2 groups: 1 group was aware of the real nature of the device, but the second group was informed only that the device aimed to assess body posture.

## Methods

### Participants

Eighty healthy participants (44 boys, 36 girls) from several primary care pediatrician offices in northern France volunteered for the study. The inclusion criteria were: (*i*) subjects were aged between 10 and 18 years old; (*ii*) informed consent was signed by the participant as well as his/her parents; (*iii*) absence of medical contraindication for daily PA (e.g. cardiovascular diseases, musculoskeletal pain); and (*iv*) no simultaneous inclusion in other biomedical studies. All participants underwent a medical examination to exclude potential contraindications for the study.

Before the study began, the aims and objectives were explained carefully to each adolescent and their parents. Written informed consent was obtained from the adolescent and the parents. Written informed consent was obtained from the adolescent and the parents. The study was approved by the Research Ethics Committee of the University of Lille (Comité Protection des Personnes, Nord Ouest IV, Lille, France). All procedures were performed according to the ethical standards of the Helsinki Declaration of 1975, as revised in 2008, and European Good Clinical Practice.

### Procedure

**Fig. 1:**
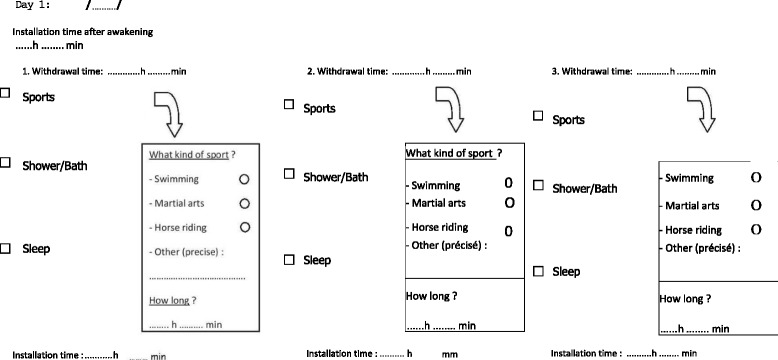
The log diary used in this study

The study started in September 2013 and ended in June 2015. Participants were referred to the Clinical Research Centre of the Lille University Hospital. Body mass index was measured to the nearest 0.1 kg using an electronic scale (Seca, Hamburg, Germany) after removal of shoes or heavy outer garments. Height was measured to the nearest 0.1 cm using a stadiometer (Seca). The included participants were randomized using sealed envelopes between the blinded group (*n* = 40) and the nonblinded group (*n* = 40). The randomization was centralized in the study center using a 1:1 ratio of blinded to nonblinded participants, which was divided into height blocks to obtain 4 blinded participants and 4 nonblinded participants per block. Participants assigned to the blinded group were informed that the study targeted the reliability of a new device assessing body posture. However, participants assigned to the nonblinded group were aware of the device being an accelerometer for PA assessment. The participants in the nonblinded group were given full information about the device, i.e. how the device detects body movements, measures sedentary behaviors (e.g. sleeping, lying before the TV, playing video games) as well as vigorous activities (e.g. playing soccer, cycling, running). The explanations were given by the same researcher, who was not blinded about the randomization of the groups. Participants wore the accelerometer on their lower back under their clothing using an elastic belt and adjustable buckle. All participants were instructed to remove the device during contact sports, water-based activities (swimming, showering, and bathing), and overnight. To fairly assess PA patterns in both groups, the participants were asked to keep a log diary of when and why the device was removed (Fig. 1) [14]. The sport activities performed after removal of the device were classified in agreement with the Compendium of Energy Expenditures for Youth and time spent in moderate or vigorous PA was added. Activities between 4 and 6 metabolic equivalents of task (MET) were defined as moderate PA and activities with 6 or more MET were defined as vigorous PA [15]. According to consensus recommendations for assessing PA in youth, a minimum of 2 or 3 days measured is needed to estimate weekly usual PA behavior in children and adolescents [16–18]. We a priori decided to record 4 days of PA behavior to ensure that our data reflected weekly PA, including leisure time. The accelerometers recorded activity for 4 consecutive days (2 school days and 2 school-free days) in free-living conditions. The devices were collected after the 4-day monitoring and the data were transferred from the device to a computer. To ensure the compliance of the subjects of the blinded group, the following 3 questions were asked: (*i*) Did you search online for any information regarding the device? (*ii*) Did you receive any information about this device by your peers? If so, which? (*iii*) Are you aware of the use or utility of this device?

### Materials

The triaxial accelerometer used was the ActiGraph® Monitor (Model GT3X; ActiGraph, Pensacola, CA, USA) (46 × 33 × 15 mm; weight 19 g, additional technical features) [19]. The accelerometer measures acceleration and deceleration in 3 spatial dimensions according to a vertical vector (x), an anteroposterior vector (y), and a mediolateral vector (z). The vector magnitude (VM) was calculated as follows: VM = √(×2+y2+z2). The epoch interval for the accelerometer was set at 1 s. A computer was used to initialize and synchronize the accelerometer. Participants who recorded less than 10 h of activity per day were excluded from the analyses [18]. PA levels were categorized as follows: sedentary activity, 0–180 counts.15 s^−1^; light activity, 181–757 counts.15 s^−1^; moderate activity, 758–1112 counts.15 s^−1^; and vigorous activity, >1112 counts.15 s^−1^ [20]. The interinstrument reliability of this device is reported to be better for moderate and vigorous activities than for sedentary activity [19, 21]. Data were averaged and expressed in counts.min^−1^.

### Outcomes

The primary outcome was the overall total counts per minute over the 4 day-period. The secondary outcome was the daily time spent at each PA level (sedentary, light, moderate, vigorous, and moderate to vigorous) calculated over the 4 day-period. All outcomes were also analyzed over the 4-day period, and school days were separated from school-free days.

### Sample calculation and statistical analysis

Based on the data of Martinez-Gomez and colleagues, we hypothesized that the mean of total counts.min^−1^ would be reduced by 20% in the blinded group compared with the nonblinded group (corresponding to an absolute difference of 100 counts counts.min^−1^) [22]. With a standard deviation of 150 counts.min^−1^, a power of 80% and an alpha risk of 5%, 37 adolescents per group were required to detect this effect size (effect size of −0.67 considered as medium to large according to Cohen) [23]. To compensate for the potential missing data, inclusion of 40 subjects per group was required. Data were analyzed using SAS software (version 9.3; SAS Institute, Cary, NC). Statistical testing was conducted at the 2-tailed α-level of 0.05. Data were expressed as mean ± standard deviation (SD) or percentages as appropriate. Normality of distributions was assessed using histograms and the Shapiro–Wilk test. Data between groups were compared using Student’s *t* test. Cohen’s d effect sizes (standardized mean differences between nonblinded vs. blinded groups) were calculated and interpreted as small for absolute value = 0.2, medium for value = 0.5, large for value = 0.8 and very large effect for value = 1.3 [23].

## Results

Eighty participants were enrolled in the study and equally randomized into 2 groups. Two teenagers (aged 14 and 16 years old) assigned to the blinded group were excluded because of missing data in PA assessment (monitoring failure). The 38 participants from the blinded group remained unaware of the true nature of the device (according to data from the ad hoc questionnaire) and their main characteristics (Table 1) overlapped with those of the nonblinded group (*n* = 40).

**Table 1:** Main characteristics of the youngsters included in the study

	All (*n* = 80)	Blinded group (*n* = 38)	Non-blinded group (*n* = 40)	p
Boys/Girls, n (%)	45 (57.7) / 33 (42.3)	21 (55.3) / 17 (44.7)	24 (60.0) / 16 (40.0)	0.67
Age (*yr*)	13.1 ± 2.3	13.5 ± 2.4	12.8 ± 2.1	0.22
Weight (*kg*)	46.2 ± 12.4	48.0 ± 13.0	44.5 ± 11.6	0.21
Height (*cm*)	156.4 ± 13.7	158.6 ± 14.9	154.3 ± 12.3	0.17
Body mass Index (*kg/m* ^*2*^)	18.6 ± 2.8	18.7 ± 2.5	18.4 ± 3.0	0.66

Values are mean ± Standard deviation

The duration of wearing the accelerometer did not differ between the 2 groups for the overall 4-day-period, nor did it differ after separating school days from school-free days. Similarly, total counts per day over the 4 day-period did not vary significantly between the nonblinded and blinded groups (mean ± SD, 178.8 ± 57.9 vs. 201.8 ± 69.5; effect size, −0.36, *p* = 0.12) (Table 2). Only 3 participants performed PA during sports (i.e. swimming, contact sports) after removal of the accelerometer, as reported in their log diaries. This accounted for 180 min out of the 60,366 min of the total recording (0.02%). This small amount of time was considered irrelevant. Table 2 details the PA patterns, including all activities. No difference was observed between school days and school-free days on any of the PA parameters between the 2 groups (Table 2).

**Table 2:** Physical activity patterns according to the awareness of the accelerometer

	Blinded group (*n* = 38)	Non-blinded group (*n* = 40)	Effect size (P)
All days			
Daily total counts	178.8 ± 57.9	201.8 ± 69.5	−0.36 (0.12)
Overall wearing duration, min	712.0 ± 81.3	737.5 ± 85.8	−0.30 (0.18)
Time spent in sedentary PA, %	73.0 ± 8.1	69.4 ± 9.1	0.42 (0.07)
Time spent in light PA, %	19.4 ± 6.2	21.8 ± 6.3	−0.38 (0.09)
Time spent in moderate PA, %	4.7 ± 2.3	5.5 ± 3.0	−0.30 (0.20)
Time spent in vigorous PA, %	2.9 ± 1.8	3.3 ± 2.1	−0.20 (0.42)
Time spent in MVPA, %	7.6 ± 3.3	8.8 ± 4.2	−0.32 (0.19)
School days			
Daily total counts	194.4 ± 64.7	207.3 ± 70.1	−0.19 (0.40)
Overall wearing duration, min	779.6 ± 94.9	800.9 ± 97.8	−0.22 (0.33)
Time spent in sedentary PA, %	71.7 ± 8.4	68.8 ± 9.4	0.32 (0.16)
Time spent in light PA, %	19.6 ± 6.5	22.3 ± 6.8	−0.41 (0.07)
Time spent in moderate PA, %	5.2 ± 2.3	5.5 ± 3.3	−0.10 (0.63)
Time spent in vigorous PA, %	3.6 ± 2.4	3.4 ± 2.2	0.09 (0.75)
Time spent in MVPA, %	8.8 ± 3.8	8.9 ± 4.4	−0.02 (0.88)
School-free days			
Daily total counts	165.0 ± 66.6	196.3 ± 88.2	−0.40 (0.08)
Overall wearing duration, min	643.9 ± 115.4	674.1 ± 104.2	−0.28 (0.24)
Time spent in sedentary PA, %	74.1 ± 9.6	70.0 ± 11.0	0.40 (0.09)
Time spent in light PA, %	19.3 ± 6.7	21.4 ± 7.3	−0.30 (0.20)
Time spent in moderate PA, %	4.3 ± 3.4	5.5 ± 3.7	−0.34 (0.14)
Time spent in vigorous PA, %	2.3 ± 1.8	3.2 ± 2.8	−0.38 (0.12)
Time spent in MVPA, %	6.6 ± 4.5	8.6 ± 5.4	−0.40 (0.08)

## Discussion

The present study is the first to examine whether children and adolescents changed their PA behavior when wearing motion sensors using an experimental design. Our findings clearly showed that there was no difference in PA patterns (school days and school-free days) between the young people in the blinded vs. the nonblinded groups, which contrasted with what we had hypothesized initially.

Two previous studies addressing this question arrived at contrasting results, but the authors used an indirect methodology [6, 8]. Instead of randomizing the children into different groups according to their awareness of the accelerometer, the researchers compared the measurements of the first day to the 6 following days [6, 8]. In the first study, the authors observed a 3–7% reduction in PA over the 6 remaining days compared to the first day, which evoked the presence of reactivity to the accelerometer in children [6]. In the second study, Davis and Loprinzi showed no evidence of reactivity in US children and adolescents when considering the total activity counts per day or the time spent in MVPA [8]. Davis and Loprinzi described the reasons for the discrepancy using results from Dösseger et al., i.e. the population differences and the structure of the built environment that may elicit more engagement in free-play situations for Swiss children than that for their American counterparts [6, 8]. The methodological differences and biases induced by the awareness of the nature of the device by all patients can justify the disagreement in findings between these 2 studies and ours [6, 8].

Other studies using a pedometer in adults mostly suggest reactivity [24–26]. However, several factors should be considered. Firstly, adults are more sensitive to the current PA recommendation and their own PA compared to children and adolescents [27, 28]. Secondly, some teenagers cannot do PA when they wish because of their family constraints, lifestyles, and/or school rhythms [29, 30]. Another interesting difference can be observed in the direct and continuous view of the results; adults could observe the digital screen of their pedometer, which contrasts with the measurement of PA by the accelerometer in our study, where all subjects remained blind to their individual counts throughout the study. In addition, when PA was assessed by use of a pedometer, the participants were asked to file a daily step log, which may have further affected their motivation for PA. Wearing a pedometer has been reported to be a simple, noninvasive way to increase awareness of daily activity, which led to an increase in PA by adult women [31].

The main strength of the study is the use of the random assignment of treatment condition vs. real covert condition, which provides confidence in our findings. However, our results have to be interpreted with caution because we only included healthy young people. Therefore, our conclusions cannot be extended to pathologic conditions. It would be judicious to investigate the reactivity in subpopulations including overweight or obese children and other chronic diseases such as respiratory diseases or diabetes. Finally, we did not collect the parents’ education level or socioeconomic status. These parameters may influence attitudes to healthy lifestyles, PA, and the motivation to wear a monitor.

## Conclusions

Our findings show that the awareness of wearing an accelerometer does not influence PA and PA patterns in healthy youth people. Therefore, the study confirms that accelerometry is an objective method that accurately reflects habitual PA and can be used for monitoring PA in children and adolescents.

## Abbreviations

MVPA: Moderate-to-vigorous physical activity
PA: Physical activity
